# Protocol for observing tunneling nanotube formation and function in both fixed and live primary mouse neurons and microglia coculture system

**DOI:** 10.1016/j.xpro.2025.103723

**Published:** 2025-04-03

**Authors:** Vivian Baker, Dimitri Budinger, Sean-Patrick Riechers, Michael T. Heneka

**Affiliations:** 1 Luxembourg Centre for Systems Biomedicine, University of Luxembourg, 6 Avenue du Swing, 4367 Esch-Belval, Luxembourg

**Keywords:** Cell Biology, Flow Cytometry, Microscopy, Neuroscience

## Abstract

Microglia and neurons can connect via tunneling nanotubes (TNTs), facilitating the transfer of organelles, vesicles, and proteins. Here, we present a protocol for visualizing murine TNT formation and material transfer between neurons and microglia in both fixed samples and samples for live-cell imaging, as well as for flow cytometry. We describe steps for identifying and measuring TNTs and quantifying the transport of aggregated proteins, such as α-synuclein or tau, between these cells.

For complete details on the use and execution of this protocol, please refer to Scheiblich et al.^1^

## Highlights

• Identification of TNT formation between neurons and microglia in live microscopy
• Quantification of protein aggregates and organelles through TNTs using flow cytometry
• Analysis of TNT morphological parameters between neurons and microglia

## Abstract (editor highlights)

Publisher’s note: Undertaking any experimental protocol requires adherence to local institutional guidelines for laboratory safety and ethics.

## Graphical abstract

**Figure:**
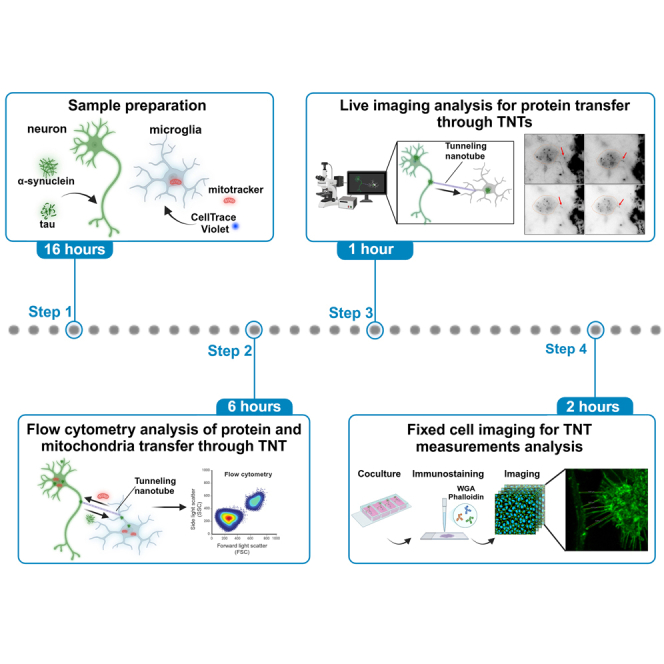

## Before you begin

Tunneling nanotubes (TNTs) are defined as thin and transient cellular structures that allow direct cytoplasmic connections between at least two cells.^2^ These cellular projections arise from the rearrangement of actin filaments, enabling continuity between cells. *In vitro*, TNTs are characterized by their formation above the cellular substratum, their extremely thin structure (measuring only a few micrometers in length and a few nanometers in width), and their ability to facilitate the transfer of proteins, organelles, virus, and ions between cells.^3^ In neurodegenerative disorders like Parkinson’s disease and Alzheimer’s disease, TNTs have been implicated in the transfer of toxic protein aggregates between neurons and microglia, as well as the exchange of mitochondria to support survival in burdened cells. Investigating the role of TNTs in the progression of neuropathologies will provide valuable insights into the health of neurons and microglia in these disorders.

The protocol below describes the specific steps for using primary murine cortical neurons and microglia to investigate TNT formation between these cells. However, we have also adapted this protocol in iPSC-derived neurons and microglia, as described in Scheiblich et al., 2024.^1^ Primary neuronal cultures were prepared from embryonic pups (E15-16) taken from pregnant C57BL/6J mice, as described in Scheiblich et al., 2024.^1^

The density of neuronal culture seeding need to be decided at the time of culture preparation. For transfer experiment described in Preparation One, 8 × 10^4^ cortical neurons are seeded in 24-well plates. For fixed and live-imaging experiments as described in Preparation Two and Three, are seeded in μ-Slide 8 wells at a density of 4 × 10^4^ cells per well. All cell carriers require Poly-D-lysine coating for 16 h at 100 μg/mL concentration to ensure proper cell adhesion. Seeded neurons are kept in complete neuronal media culture until DIV14, when they are at the optimal time point for experiment. In the same paper by Scheiblich et al., 2024, the steps outlined to obtain primary microglial cultures from neonatal C57BL/6J pups (P0–P3) are also described. In short, microglia are allowed to grow in a mixed-glial culture conditions in complete microglial media culture in 5 μg/mL Poly-D-Lysine coated T-75 flasks. DIV14 microglia are collected from mixed culture by mechanical separation (orbital shaking) and seeded in 6-well plates with a density of 2 million cells per well in experimental microglial culture medium to recover for 16 h. After being stained according to Preparation One, microglia are seeded onto neurons with a density of 1.25 × 10^5^ cells per well for transfer experiment and 5 × 10^4^ cells per well for live imaging and immunocytochemistry experiments.

Recombinant α-synuclein and tau fibrils were kindly donated by our collaborator (R. Melki). Details for this preparation can be found in Scheiblich et al., 2024^1^ and here.^4^^,^^5^^,^^6^^,^^7^^,^^8^ In our protocols, the recombinant proteins are used to induce burdens in the neurons, who “donate” said fibrils to microglia in TNTs-mediated transfers. Hence, the neurons are also known throughout the protocols as “donors” or “donor cells”, and the microglia are known as “acceptors” or “acceptor cells”.

Prior to beginning these experiments, key resources (listed in the key resources table) should be available.

### Institutional permissions

All procedures performed in this study using laboratory animals were in accordance with the Grand-Ducal Regulation of 11 January 2013 on the protection of animals used for scientific purposes as well as the Act of 27 June 2018 on the protection of animals, which are in concert with the European Communities Council Directive of September 22, 2010 (2010/63/EU). *Post-mortem* isolation of primary cells from non-burdened wild-type pubs (C57BL/6J) does not require further project approval from the Animal Experimentation Ethics Committee (AEEC) of the University of Luxembourg and the Luxembourgish Ministries of Agriculture and of Health.

Animal care and handling were performed according to the animal welfare requirements of the Luxembourgish Ministry of Agriculture.

### Preparation one: Cell preparation for α-syn, tau, and mitochondria transfer experiments using flow cytometry

**Timing: ∼24 h**

**Timing: 16 h, hands on: 15 min (step 1)**

**Timing: 30 min (step 2)**

**Timing: 6 h, hands on: 20 min (step 3)**

**Timing: 1.5 h (step 4)**

1. Fibril loading and labeling of neurons (donor cells):
  a. In complete neuronal medium, prepare 0.1 μM ATTO-488-labeled α-synuclein fibril or 0.1 μM ATTO-488-labeled tau fibril solution.
  b. Replace neuronal medium with prepared α-synuclein or tau fibril solution.
  c. Incubate DIV14 neurons in an incubator (5% CO_2_, 37°C) for 16 h.
  d. After incubation, remove the fibril solution, and wash the neurons twice with complete neuronal media to remove excess fibrils.
  e. For mitochondria transfer experiment:
    i. in 1X PBS, prepare 1:1000 dilution of 5 mM stock solution of CellTrace Violet to a 5 μM working concentration.
      ***Note:*** Stock solution of Cell Trace Violet can be prepared by dissolving the reagent in provided DMSO per manufacturer’s protocol.
    ii. Remove neuronal medium and wash the cells once with PBS to remove traces of medium.
    iii. Incubate the cells with prepared labeling solution in the incubator for 10 min.
    iv. Add DMEM containing Fetal Bovine Serum (FBS) to remove excess dye free floating in the solution.
      **CRITICAL:** To successfully remove free dye, the culture medium used, regardless of cell type, should always contain at least 1% protein such as FBS.
    v. Remove the solution and incubate neurons again in neuronal culture medium.
      **CRITICAL:** Once thawed, Atto-488 α-synuclein and tau fibrils cannot be refrozen or kept at 4°C but remain stable at 22°C for 48 h. These fibrils are light sensitive and must be kept in the dark.
2. Microglia staining and labeling of mitochondria (acceptor cells):
  a. Labeling and preparation of microglia.
    i. For aggregate transfer experiment: in 1X PBS, prepare 1:1000 dilution of 5 mM stock solution of CellTrace Violet to a 5 μM working concentration.
    ii. For mitochondria transfer experiment: in 1X PBS, prepare 1:2000 of 1 mM stock solution of MitoTracker Red CMXRos to a 500 nM working concentration.
      ***Note:*** Stock solution of MitoTracker Red CMXRos can be prepared by dissolving the reagent in the provided DMSO according to manufacturer’s protocol.
  b. Remove microglial medium and wash the cells once with PBS to remove traces of FBS.
  c. Incubate the microglia with prepared labeling solution in the incubator for 10 min.
  d. Dilute free floating dye by adding microglia medium containing FBS.
  e. Wash microglia three times with PBS to remove traces of FBS and to remove excess MitoTracker dye (in mitochondria transfer experiment).
  f. Detach microglia by incubating the cells with Trypsin 0.5% at 5% CO_2_, 37°C for 5–10 min until the cell layer detach.
  g. Centrifuge the cells at 300 g for 5 min at 22°C and resuspend the pellet in desired volume in a conical Falcon tube.
    ***Note:*** Different combinations of colors for Cell Trace and MitoTracker can be used depending on the dyes’ colors of the fibrils. For a complete list of available Cell Trace kits and MitoTracker kits can be found on the website of Thermo Fisher Scientific.
3. Co-culturing of neurons and microglia:
  a. Seed microglia at a density of 1.25 × 10^5^ cells per well on top of seeded neurons for 0–6 h in the incubator to allow for TNT-mediated transfers to occur over the time course.
  b. As negative controls, TNTs formation is inhibited between donors and acceptors by both mechanical and chemical inhibitions. For mechanical inhibition, microglia are prevented from direct cell-to-cell contact by being seeded in 3 μm Transwell hovering on top of neurons. Conversely, TNTs formation is inhibited between microglia and neurons by using 0.5 μM cofilin inhibitor Cytochalasin D (CytD) for 0–6 h.

**CRITICAL:** Enough wells of neurons need to be generated to accommodate the desired time points, compensation controls, and technical replicates for the experiment. I.e. microglia can be added to neurons every hour, generating 7 time points (0, 1, 2, 3, 4, 5, 6^th^ h) or every three hours, generating 3 time points (0, 3, 6^th^ h). For example, for two replicates, 3 time points, and 3 compensation controls (unstained and singled stained – green and red), at least 9 wells of neuronal cultures need to be generated. While waiting to be seeded on the neurons at the right time points, microglia need to be kept on ice in the dark to protect cell viability and the fluorescent dyes.

4. Cell collection and flow cytometry measurements:
  a. After 6 h, detach co-cultured, Transwell, and CytD-treated cells with Accutase in the incubator for 10 min.
  b. Dilute Accutase in 1:5 ratio in DMEM containing FBS, and gently use a P1000 pipet tips to lift and flush the detached cell layer from the wells and collect the cells into round-bottom polystyrene test tubes specifically for flow cytometry.
  c. Centrifuge the cells at 300 g for 5 min at 22°C.
  d. Resuspend the cell pellets in 300 μL ice-cold PBS + 2% BSA.
  e. Measure transfers by flow cytometry using a suitable BD FACS machine, i.e., FACS CANTO II or FACS Fortessa and BD FACSDiva software. Specific filter/laser requirements for readout measures can be found in this step-by-step method details section.
  f. Cell-to-cell transfer of α-synuclein and/or mitochondria are quantified using FlowJo.

### Preparation two: Neuronal-microglia cocultures labeling and live-imaging

**Timing: up to 20 h**

**Timing: 16 h, hands on: 15 min (step 5)**

**Timing: 15 min (step 6)**

**Timing: 10 min (step 7)**

**Timing: variable (step 8)**

5. Neurons α-synuclein or tau loading (donor cells):
  a. Thaw out at 22°C a vial of Atto-488-labeled α-synuclein or Atto-488-labeled tau fibrils. For α-synuclein and tau fibrils preparation, refer to Scheiblich et al. (2024).
  b. Resuspend the Atto-488-labeled α-synuclein or Atto-488-labeled tau fibrils in complete neuronal medium to a final concentration of 0.2 μM.
  c. Take neurons out of the incubator and replace medium with freshly prepared 0.2 μM α-synuclein or tau fibrils medium.
  d. Incubate neurons in a CO_2_ incubator at 37°C for 16 h.

***Note:*** During this period, neurons will internalize the Atto-488-labeled α-synuclein or Atto-488-labeled tau fibrils, resulting in the appearance of fluorescent particles within the soma and neurites when observed under a microscope.

**CRITICAL:** Once thawed, Atto-488 α-synuclein and tau fibrils cannot be refrozen or kept at 4°C but remain stable at 22°C for 48 h. These fibrils are light sensitive and must be kept in the dark.

**Figure 1:**
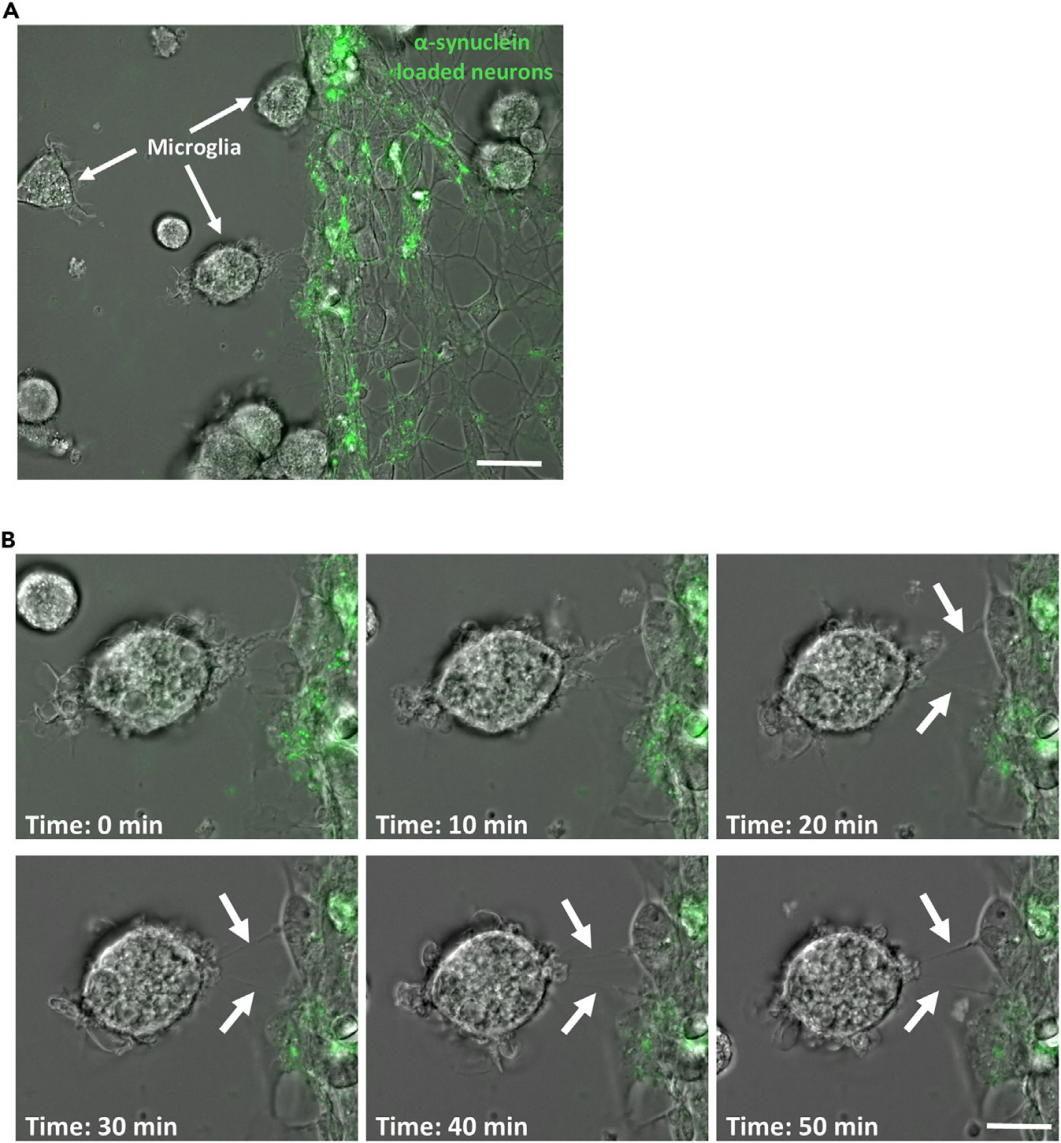
Time-lapse imaging for TNT formation between neurons and microglia (A) Example of area of interest for live-imaging experiment. Neurons are loaded with ATTO-488 α-synuclein and microglia are plated on top of the neuronal culture. An area where neurons and microglia are in close proximity maximizes the chances to observe TNT formation between these cells. Scale bar is 10 μm. (B) Example of time-lapse analysis showing TNT formation between neurons and microglia. White arrows point to TNTs between these cells. Scale bar is 5 μm.
  c. Set up Z-stacks recordings with between 0.2 – 0.5 μm steps per stack.
    ***Note:*** Stacks should be set from the bottom of the cell (close to substratum) up to the top of the cells, to maximize capturing free-floating TNT formation between these cells. Depending on cell thickness, each complete stack may consist of 5 to 10 images.
  d. Acquire time lapse movies maintaining the temperature at 37°C and 5% CO_2_ during recordings.
    ***Note:*** We found the acquisition to be optimal using a 40X water immersion objective, with one frame recorded every minute.
  e. Observe the formation of TNTs between neurons and microglia over a period of 30 min until a few hours, depending on the stability of the cells under microscopy settings (Figure 1B).
  f. Following acquisition, analyze videos using FIJI (ImageJ) (see “Live-cell imaging analysis: Identification of TNT formation and protein transfer between neurons and microglia” section) to quantify TNT length, α-synuclein or tau total travel distance, mean velocity, and total time to travel from neuron (donor) to microglia (acceptor) cells.
    ***Note:*** Not all microglia and neurons in the recording field will generate TNTs. It is therefore advisable to acquire different fields of view using a tile scan mode during recordings, to maximize catching TNT formation between these cells. Video recordings can vary between 1 h to 3 h, depending on the stability of the cultures under imaging settings and the stability of TNTs during recording.
    **CRITICAL:** Microglia are sensitive to high laser power and tend to burst over time. It is therefore crucial to lower the laser intensity to a minimum during acquisition. We found that a laser intensity set between 20% and 40% and an exposure time of around 10 ms to be optimal and reduce photosensitivity of cells and dyes during recordings. Additionally, TNTs are photosensitive and can easily break due to light flashes during recordings. We found an acquisition rate of one image per minute to be optimal for minimizing photosensitivity in TNTs as well as photobleaching of the ATTO-488-labeled α-synuclein and tau fibrils.

6. Microglia collection (acceptor cells):
  a. Wash microglia once with DPBS to remove excess medium.
  b. Add 0.5% trypsin to microglia and incubate in the CO_2_ incubator at 37°C for 5 min.
    i. Microglia will start to change shape and become rounder.
    ii. After 5 min, detaching microglia will become brighter and some start to detach.
  c. Gently detach microglia by flushing them using a P1000 pipette and transfer to a 15 mL falcon tube containing DMEM supplemented with 10% FBS.
    ***Note:*** Microglia are sometimes difficult to detach and require more time to detach (up to 8 min). In case microglia do not detach with the P1000 pipette, scrape the remaining cells using the end of a P200 tip to detach adherent cells.
  d. Collect 10 μL of cell suspension to estimate viable cell number using a cell counting chamber.
  e. Spin the microglia at 300 g for 5 min.
  f. Resuspend the pellet in the desired volume of microglia medium.
7. Microglia – neuron coculture.
  a. Wash the neurons with DPBS twice to remove any fluorescent α-synuclein or tau fibrils that were not internalized into the neurons.
    ***Note:*** Neurons can detach during these washing steps. It is important to always remove and add medium from the same corner of the culture dish with care to avoid detachment.
  b. Seed 5 × 10^4^ microglia onto neurons, let the microglia settle for 5 min at 37°C, and start live imaging recording immediately.
    **CRITICAL:** Microglia are plated onto neurons immediately before recordings to enable the visualization of TNT formation dynamics between these cells. Allowing the cells to settle for a few hours instead may hinder the observation of these structures as they form.
8. Live imaging for TNT formation and protein transfer.
  a. Live imaging is performed using a microscope allowing both fluorescence and bright-field imaging.
    ***Note:*** In this protocol, we describe the use of the Zeiss Axio Observer 7 microscope for live-imaging. However, we have also used the following microscopes for the same purpose: the Nikon Elipse Ti-E microscope and the Zeiss Celldiscoverer 7 microscope.
  b. Spot an area where microglia are clearly identifiable in close proximity to ATTO-488- α-synuclein labeled neuron (Figure 1A).
    **CRITICAL:** Microglia appear round at this stage, as they were detached and plated onto neurons just before recording. Within minutes, they begin adhering to the bottom of the well, gradually transitioning into a more elongated and flattened morphology. As motile cells, microglia can migrate across the culture dish, potentially forming TNTs with stationary neurons. In contrast, neurons are easily identifiable by their elongated structure, characterized by an interconnected network of oval cell bodies (somas) and intricate neurites. In this specific culture setup, neurons are also fluorescent, as they have been loaded with ATTO-488-α-synuclein, enabling clear visualization.

### Preparation three: Immunostaining of fixed neuronal-microglia cocultures

**Timing: up to 24 h**

**Timing: 16 h, hands on: 15 min (step 9)**

**Timing: 15 min (step 10)**

**Timing: 6 h (step 11)**

**Timing: 2 h (step 12)**

**Timing: variable (step 13)**

9. Preparation of neurons (donor cells):
  a. Neurons are loaded with ATTO-488-labeled α-synuclein or tau fibrils as per instructed in preparation two, step 5 of this protocol.
  b. Incubate neurons in a CO_2_ incubator at 37°C for 16 h.
10. Labeling of microglia (acceptor cells):
  a. Wash microglia once with DPBS to remove excess medium.
  b. Depending on fluorophore used, prepare a CellTrace Violet or CellTrace Deep Red solution.
    i. Resuspend CellTrace dye in the appropriate volume of DMSO as per manufacturer’s instructions (Thermo Fisher Scientific).
    ii. Dilute the CellTrace dye stock solution in DPBS, to a 2.5 μM working solution.
  c. Stain microglia with CellTrace dye by incubating cells for 10 min in a CO_2_ incubator at 37°C.
  d. Remove dye solution, wash with DPBS, and collect microglia as per instructed in preparation two, step 6 (“Microglia collection (acceptor cells)”) of this protocol.
11. Microglia-neuron coculture:
  a. Wash the neurons with DPBS twice to remove any fluorescent α-synuclein or tau fibrils that were not internalized into the neurons.
  b. Seed 5 × 10^4^ microglia onto neurons in microglia medium.
  c. Incubate the coculture in a CO_2_ incubator at 37°C for 6 h.
12. Immunostaining:
  a. Remove coculture medium and wash twice with DPBS.
  b. Fix the cells with ice cold 4% PFA dissolved in DPBS for 10 min at 22°C.
  c. Block and permeabilize the cells with 5% normal goat serum dissolved in DPBS, supplemented with 0.1% Triton X-100 for 45 min at 22°C.
  d. Wash twice with DPBS.
  e. Incubate with fluorescent dyes diluted in DPBS for 1 h at 22°C, at the following working concentrations: Wheat Germ Agglutinin (WGA) coupled to Alexa Fluor 488 for membrane staining (0.01 mg/mL) and Phalloidin coupled to Alexa Fluor 568 for F-Actin staining (50 nM).
  f. Wash three times with DPBS.
  g. Keep the stained μ-Slide 8 wells in the dark at 4°C until image acquisition.

***Note:*** If the cells are cocultured on a glass coverslip, these can be mounted on a slide using a few drops of Dako Fluorescence Mounting Medium. In this case, let the mounting medium sit for at least 24 h at 22°C in the dark prior to imaging.

**CRITICAL:** All along immunostaining steps, take extra caution not to flush the cultures or cause excess movements of the slides as TNTs are very fragile and mechanical stress can easily break them.

**Figure 2:**
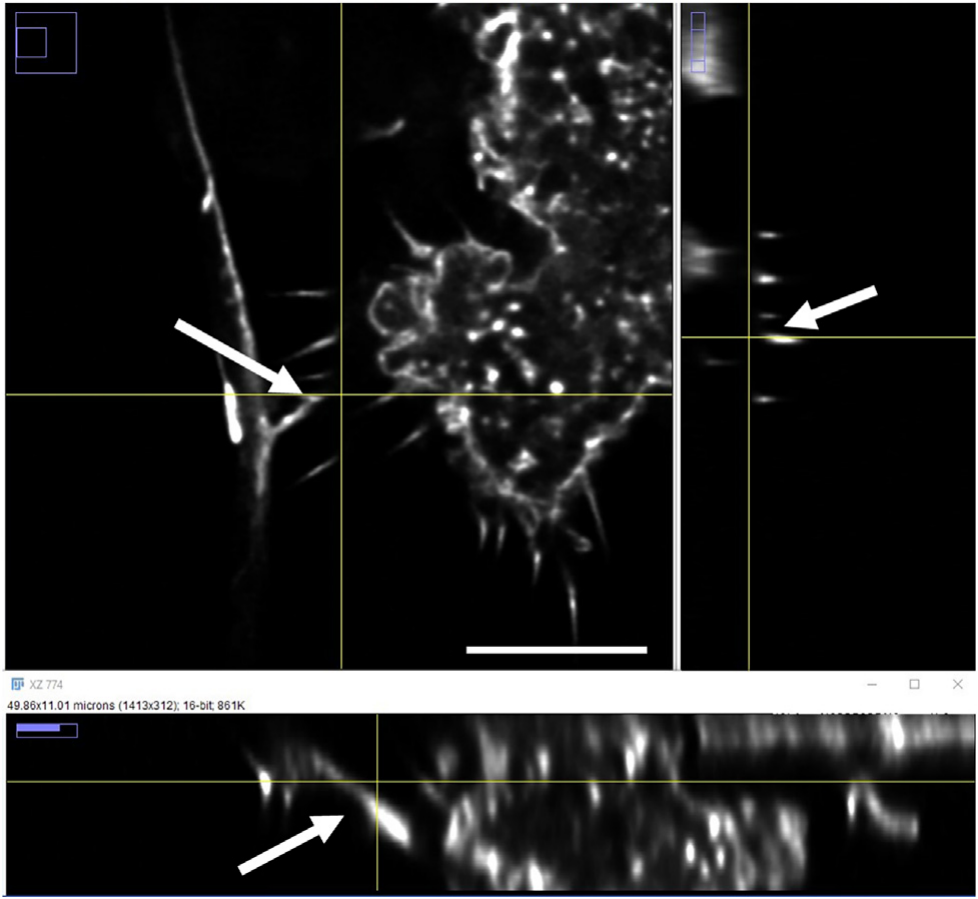
Orthogonal projection analysis for TNTs identification between neurons and microglia This analysis can be performed using ImageJ, using the following options: Image > Stacks > Orthogonal views, or use the Ctrl+Shift+H shortcut. The intersection of the yellow lines represents the point in the stack that is being analyzed, and allows to display the XZ and YZ planes at a given point in the 3D image. Here, we show that TNTs are free-floating structures, that do not touch the substratum. White arrow points to a free-floating TNT. Scale bar is 5 μm.
  d. Following acquisition, analyze images using FIJI (ImageJ) (see method) to quantify parameters such as TNTs length, number of TNTs per cells, tube height above substratum, and the presence of α-synuclein or tau fibrils within TNTs.

13. Image acquisition:
  a. Acquire images using a confocal microscope.
    ***Note:*** We found the acquisition to be optimal using a confocal microscope that allows higher resolution images. For instance, we have used the Leica SP8 LIGHTNING confocal microscope or a Zeiss LSM 800 or 900 confocal microscope. Acquisition with 0.035 μm × 0.035 μm x 0.250 μm scaling per pixel, the laser power below 2% and a frame time of around 2 s gave optimal resolution images of TNTs.
  b. A minimum 63X oil immersion objective should be used to better visualize TNTs.
  c. Take images using Z-stacks with around 0.2–0.5 μm steps per stack to identify TNTs.
    **CRITICAL:** By definition, TNTs are free-floating cellular protrusions that connect two cells without touching the substratum below. TNTs are therefore distinguishable from other cellular protrusions in the coculture system (such as filopodia and neurites) by their height above the substratum, based on their Z plane within the stack. One must play with the microscopic focus wheel to check that the TNT is above the substratum and not on the bottom of the dish. This can be visualized using an orthogonal projection, which shows the TNTs free-floating characteristics (Figure 2).

## Key resources table

**Table:** 

REAGENT or RESOURCE	SOURCE	IDENTIFIER
**Chemicals, peptides, and recombinant proteins**		

Accutase	Invitrogen	Cat# 00-4555-56
Atto-488-labeled α-synuclein	Melki laboratory	N/A
Atto-488-labeled tau	Melki laboratory	N/A
B-27 Plus supplement	Gibco	Cat# A3582801
Bovine serum albumin (BSA) fraction V, 500 g	Carl Roth	Cat# 8076.3
CellTrace deep red	Thermo Fisher Scientific	Cat#C34565
CellTrace violet	Thermo Fisher Scientific	Cat#C34557
DMEM, high glucose, GlutaMAX supplement	Gibco	Cat# 61965059
Dulbecco’s phosphate-buffered saline (DPBS)	Gibco	Cat# 14190169
Fetal bovine serum (FBS)	Life Technologies	Cat# 10270106
GlutaMAX supplement	Gibco	Cat# 35050061
HBSS, calcium, magnesium, no phenol red	Gibco	Cat# 14025092
MitoTracker Red CMXRos dye, for flow cytometry	Invitrogen	Cat# M46752
N-2 supplement	Gibco	Cat# 17502048
Neurobasal Plus medium	Gibco	Cat# A3582901
Penicillin-streptomycin (10,000 U/mL)	Gibco	Cat# 15140122
Phalloidin Alexa Fluor 568	Thermo Fisher Scientific	Cat#A12380
Poly-D-lysine 0.1 mg/mL	Gibco	Cat# A3890401
Triton X-100	Sigma-Aldrich	Cat# X100
Trypsin-EDTA (0.5%), no phenol red	Thermo Fisher Scientific	Cat# 15400054
UltraPure DNase/RNase-free distilled water	Invitrogen	Cat# 10977035
Wheat germ agglutinin Alexa Fluor 488	Thermo Fisher Scientific	Cat# W11261

**Experimental models: Organisms/strains**		

Mouse (*mus musculus*): C57BL/6J For neurons: embryonic pups (E15–E16) For microglia: neonatal pups (P0–P2) Gender: mixed gender	Charles River Laboratories	RRID: IMSR_JAX:000664

**Software and algorithms**		

GraphPad Prism v.9.0	GraphPad Software Inc.	RRID: SCR_002798 https://www.graphpad.com/
Fiji ImageJ, v.2.0.0-rc-69/1.52n	Wayne Rusband	RRID: SCR_002285 http://fiji.sc
FlowJo, v.10.9.0	FlowJo, LLC	RRID: SCR_008520 https://www.flowjo.com/solutions/flowjo
Imaris, v.9.2.1	Bitplane by Oxford Instruments plc	RRID: SCR_007370 http://www.bitplane.com/imaris/imaris
BD FACSDiva software, v.9.0	BD Biosciences	RRID:SCR_001456 https://www.bdbiosciences.com/

**Other**		

24-well Nunclon Delta surface plate	Thermo Fisher Scientific	Cat# 142475
5 mL polystyrene round-bottom tube^3^	Falcon	Cat# 352054
6-well Nunclon Delta surface plate	Thermo Fisher Scientific	Cat#140675
μ-slide 8 well	ibidi	Cat# 80801
Acrodisc PF syringe filters with Supor membrane, sterile, 0.8/0.2 μm, 25 mm	Pall Corporation	Cat# 4187
Dako fluorescence mounting medium	Dako	Cat#S3023
CellTrics 50 μm disposable filters	Sysmex	Cat# 04-0042-2317
Corning transwell polycarbonate membrane-cell culture inserts	Sigma-Aldrich	CLS3422
BD LSRFortessa cell analyzer	BD Biosciences	N/A
SP8 microscope	Leica	N/A
Axio Observer 7 microscope	Zeiss	N/A

## Materials and equipment

For primary microglia cell culture:

**Table:** Medium for mature and isolated microglia culture (“microglia medium”)

Reagent	Final concentration	Amount
DMEM (4.5 g/L High Glucose, Glutamax-Supplement)	98% v/v	49 mL
N2-Supplement	1% v/v	0.5 mL
Penicillin-Streptomycin	1% v/v	0.5 mL
**Total**	**100% v/v**	**50 mL**

***Note:*** Prepare fresh in 50 mL quantity that can be stored at 4°C for a maximum of 3–4 weeks.

For primary cortical neurons cell culture:

**Table:** Medium for neuronal culture: Neurobasal Plus Medium + 2% B-27 Plus + 1% N2 + 1% Glutamax Supplement + 1% Penicillin-Streptomycin

Reagent	Final concentration	Amount
Neurobasal Plus Medium	95% v/v	42.5 mL
B-27 Plus Supplement	2% v/v	1 mL
N2-Supplement	1% v/v	0.5 mL
Glutamax Supplement	1% v/v	0.5 mL
Penicillin-Streptomycin	1% v/v	0.5 mL
**Total**	**100% v/v**	**50 mL**

***Note:*** Prepare fresh in 50 mL quantity that can be stored at 4°C for a maximum of 3–4 weeks.

## Step-by-step method details

### Flow cytometry: Analysis of α-syn, tau, and mitochondria transfer between neurons and microglia

**Timing: 2 h**

This step enables the quantification of aggregates and mitochondria transfers facilitated by TNTs between neurons and microglia over a time course using principles of flow cytometry. The protocol can also be used to study the effect of TNTs stimulators or inhibitors on aggregates and mitochondria transfers. The analysis is performed using FlowJo software.

**Figure 3:**
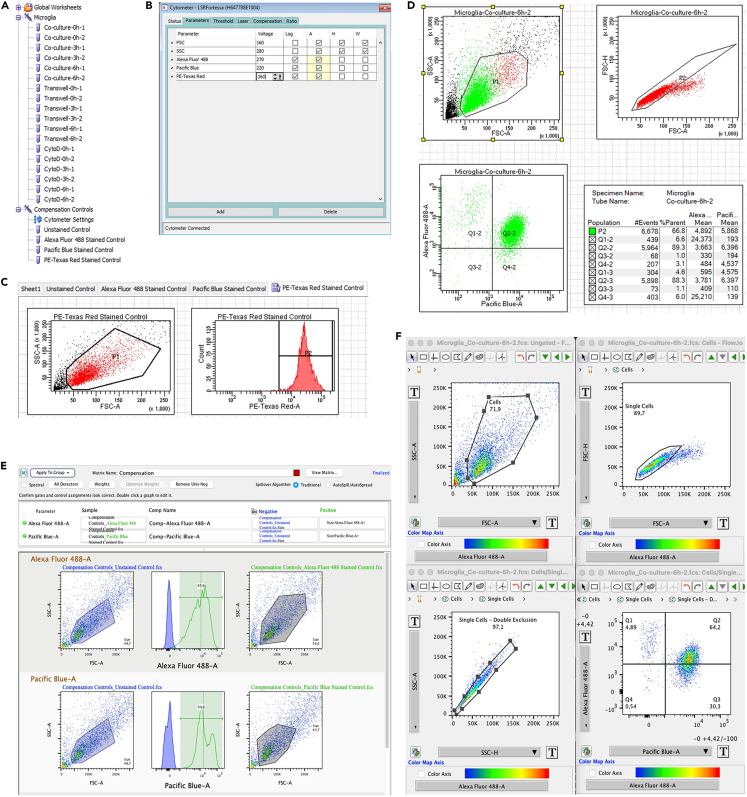
Examples of experimental set up and analysis workflow for flow cytometry experiments (A) Example of how-to setup for Specimen and Tubes on FACSDiva. (B) Example of cytometers panel setup on FACSDiva that include PMT voltage options for Forward and Side Scatter lasers, and lasers for fluorochromes of interest. (C) Example of Compensation Control panel for PE-Texas Red. (D) Example of plotting workflow and statistic display for microglia that are positive for α -synuclein after 6 h of co-culture. (E) Example of Compensation Control Setup on FlowJo for Alexa 488 and Pacific Blue. (F) Example of analysis workflow on FlowJo to quantify the number of microglia positive for α -synuclein after 6 h of co-culture.
  c. In the Cytometer panel, set up the parameters for the cytometers to include gating for all three - Area (A), Height (H), and Weight (W) for Forward Scatter (FSC) and Side Scatter (SSC) (Figure 3B).
  d. Add gating for area (A) for the three chosen fluorochromes (Figure 3B).
    i. For CellTrace Violet: 405 nm Violet Laser Excitation and DAPI/BV421/Pacific Blue 450/50 nm Emission Filter.
    ii. For ATTO-488 α-synuclein: 488 nm Blue Laser Excitation and Alexa-488/FITC/GFP 530/30 nm Emission Filter.
    iii. 561 nm Yellow-Green Laser Excitation and PE Texas-Red/mCherry 610/20 nm Emission Filter.
      ***Note:*** If different dyes are used, the user can look up the specific laser and filter according to the emission/excitation information for the dyes.
  e. Set appropriate PMT voltages for FSC and SSC to distinguish cells from debris and identify single cells.
  f. Use unstained and single-stained controls to set appropriate PMT voltages for the fluorescent channels and calculate compensation, which is the process of correcting for fluorescence spillover between channels.
    i. Go to Experiment → Compensation Setup → Create Compensation Controls.
      ***Note:*** New specimen called “Compensation Controls” will automatically appear, with new tubes defined as: Unstained Control, Alexa Fluor 488 Stained Control, BV421 Stained Control, and PE-Texas Red Stained Control. Depending on the dyes used, the program will automatically determine the names of the compensation controls.
    ii. Run each sample according to the tube name to set the voltages for each fluorescent channel. Figure 3C shows the example of gating for PE-Texas Red Stained Control created by BD FACSDiva.
      ***Note:*** The dot plot on the left allows for cells to be separated from debris, while the histogram plot on the right displays fluorescence intensity of PE-Texas Red-positive cells when the right voltage is applied.
  g. Define gating strategy in the Global Sheet for all specimens. Figure 3D shows an example of how to measure α-synuclein transfers in microglia after 6 h of incubation with neurons.
    i. Panel 1 (top left): in an SSC-A vs. FSC-A dot plot, use polygon gates to define cells population from debris. Black dots represent all events and cell population or P1 is represented by red dots.
    ii. Panel 2 (top right): in an FSC-H vs. FSC-A dot plot, use a polygon gate to define single cells population from cell doublets. Right click on this plot and select display of P1 only. Single cells or P2 are represented in the plot by green dots.
    iii. Panel 3 (bottom left): Alexa 488 vs. Pacific Blue dot plot to determine the fluorescence intensity in green and blue channels of single cells. Right click on the plot and select display of P2 only. Use the quadrant gate to determine the relative amount of single microglia which are positive for both CellTrace Violet and ATTO-488 α-synuclein.
    iv. Panel 4 (bottom right): Statistics view display the percentage of cells in each quadrant relative to the total cell count and parent population P2’s cell count. Right click on Panel 3 and select display statistics to generate Panel 4 and gate to determine the relative number of single neurons which are positive.
      ***Note:*** The same analysis can be done for mitochondria transfer by choosing Pacific Blue vs PE-Texas Red in the Panel 3 and gate to determine the relative number of single neurons which are both positive for Cell Trace Violet and MitoTracker Red.
2. Acquire a sufficient number of cells per sample on medium flow rate, aiming for around 50,000 events/sample.
3. Save experiments and FCS files for subsequent analysis with FlowJo.
4. Data analysis set up with FlowJo:
  a. Open a Workspace on FlowJo and drag the FCS files to the empty “Drag Samples Here” field.
  b. Test samples will automatically appear under All Samples and compensation controls will appear automatically under Compensation.
  c. Choose “Compensation” in the main software’s dropdown to create Compensation Matrix. A new tab called “Control Group: Compensation” will automatically appear.
  d. Figure 3E gives an example on how to create and finalize the compensation matrix for Alexa 488 and Pacific Blue.
    i. In the SSC-A vs. FSC-A dot plots of Unstained Control (left panel), Alexa-488-positive Control (right panel), and Pacific Blue-positive Control (right panel), use polygon gates to choose cell populations.
    ii. In the middle panel, use the Range gate to include only cell populations that are positive for the desired fluorochromes.
    iii. The compensation is now finalized and click on “Apply to Group” and choose All Samples. Close the Compensation tab.
  e. Analysis of samples: Figure 3F shows an example analysis of α-synuclein transfer in microglia that have been in co-culture with neurons for 6 h.
    i. Panel 1 (top left): Create an SSC-A vs. FSC-A dot plot and use the polygon gate to determine microglia population from cell debris.
    ii. Panel 2 (top right): Double click on the population, create an FSC-H vs. FSC-A dot plot and use the polygon gate to determine single cell population.
    iii. Panel 3 (bottom left): Double click on the single cell population, create an SSC-A vs. SSC-H dot plot and use the polygon gate to doubly exclude remaining cell doublets.
    iv. Panel 4 (bottom right): Double click on the double-excluded single cells population and create an Alexa-488 vs. Pacific Blue dot plot, and use a quadrant gate to determine the population of microglia that have been received α-synuclein from neurons – cell population that are positive for both CellTrace Violet and ATTO-488 α-synuclein.
    v. Statistic view is immediately available for each of the quadrant.
  f. The same analysis setup can be copied and dragged to “All Samples” to analyze the rest of the samples with reduced bias.
    ***Note:*** The same analysis can be done for mitochondria transfer from microglia to neurons by applying the same gating strategy. Instead of an Alexa 488 vs Pacific Blue dot plot for panel 4, create a PE-Texas Red dot plot to determine population of neurons that have received mitochondria from microglia.
5. Import data from FlowJo’s statistics to another statistical analysis and graphing solution like GraphPad Prism or Microsoft Excel for further analysis.

***Note:*** Examples of available analyses for this experiment include but are not limited to change or increase of transfers over the co-culture time course, how TNT-inhibitors or stimulators affect the transfers of aggregates and organelles between neurons and microglia, etc.

***Note:*** Although the plot workflow for BD FACSDiva and FlowJo are similar, and preliminary quantitative data can be gathered from BD FACSDiva, the data can only be definitively analyzed by FlowJo, which requires a valid license to operate.

1. Experimental set-up for flow cytometry:
  ***Note:*** For compensation settings, always prepare unstained controls and single-stained controls for every fluorophore used in the measurements, preferably using the same cells for experiments and not compensation beads.
  **CRITICAL:** Keep the prepared samples always on ice and in the dark before running to protect the fluorescent dyes and cell viability. Before running, cells should be filtered through a 50 μm filter to eliminate cell clumping and decrease the number of doublets.
  a. Open new experiment on BD FACSDiva software.
  b. Define your specimen and samples by adding New Specimen and adding New Tube. Figure 3A shows an example of Specimen and Tubes list for the experiment.

### Live-cell imaging analysis: Identification of TNT formation and protein transfer between neurons and microglia

**Timing: 1 h (per time-lapse movie)**

This step enables the analysis of TNTs formation dynamics by tracking changes in TNTs length between successive frames of a time-lapse movie as well as TNT persistence during this time. This step also allows the analysis of α-synuclein or tau transport dynamic through TNTs by tracking their movement along the TNT length between successive frames of the time-lapse movie. This analysis can be performed using the tools available in ImageJ software.

**Figure 4:**
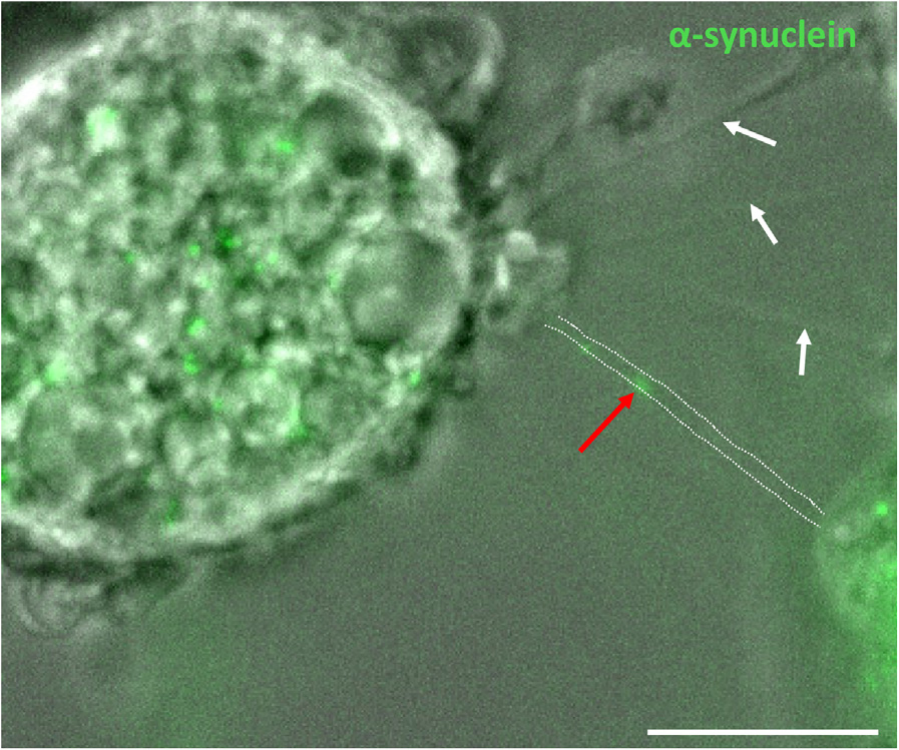
Example of live imaging showing α-synuclein transfer through a TNT Neurons were loaded with α-synuclein and incubated with microglia to promote TNT formation and protein transfer. This is a snap shot of the transfer of α-synuclein through the TNT. Dashed lines highlight a TNT with the presence of α-synuclein (red arrow). White arrows point to other TNTs visible within this frame. Scale bar is 5 μm.
  b. Split channels using ImageJ dedicated command.
  c. Using the channels tool, change the green channel (corresponding to the Atto-488 α-synuclein or tau fibrils neuronal loading) to grays.
  d. Invert LUT using ImageJ dedicated command (Figure 5).

6. TNT length measurement over time:
  a. Use ImageJ to open the movie obtained by recording neuron-microglia coculture to investigate TNT formation between these cells.
  b. In the software options, select “Image” >“Stacks” > “Z Project”.
    ***Note:*** The ZProjection window opens, with the following sub-sections: “Start slice”, “Stop slice”, and “Projection type”. By default, these values are set to the first and last slices of the stacks taken during acquisition. In “Projection type”, we found the “Max Intensity” projection to be optimal for TNT identification.
    ***Note:*** The ZProjection allows to better visualize TNTs within the stacks. In some cases, TNTs form between cells by staying on the same stack plane. In that case, the ZProjection is not specifically required.
  c. A maximum intensity Z-projection video will open in a new window, that merges all Z-stacks taken per frame into one image, but allowing for visualizing every frame of the video as well as the separate channels taken during acquisition.
  d. Image settings such as brightness and contrast as well as channels selection can be adjusted at this stage using the following options: Image > Adjust > Brightness/Contrast and Image > Color > Channels Tool.
  e. Scan through the different frames of the video until a TNT is clearly visible between neurons and microglia.
  f. Mark this frame as frame one.
  g. Use the “Freehand line” tool to draw a line along the entire length of the TNT.
  h. Measure the length of TNT using the “Analyze” > “Measure” tool, or press Ctrl+M as a shortcut.
  i. A results window opens, where TNT length is recorded.
  j. Move to the following frame and repeat the above steps to measure TNT length over time. Each measurement is recorded in a chronological order in the same results window.
7. Report the measurements and measure TNT dynamics parameters:
  a. Copy the values into an Excel file.
  b. Starting from the first frame, measure the absolute difference in TNT length with the next frame.
  c. Average all values to obtain an “average speed” of TNT formation in μm/min or μm/s, depending on the acquisition settings.
    ***Note:*** TNT formation is a very dynamic and transient phenomenon, as these structures may form then break during acquisition. Also, microglia are motile cells that feel the environment, and they may connect to different neurons via TNTs, which may vary in time and space. For these reasons, TNTs length may vary greatly over the recording time.
  d. Calculate the time of TNT persistence in min (or seconds) as the time where a TNT integrity (connecting two cells) is visible.
8. Protein transport dynamic measurements:
  a. Observe the video for TNT formation and the presence of Atto-488 α-synuclein positive signal within a TNT (Figure 4).
    **CRITICAL:** Scan through the whole stacks in each frame as carefully as possible to identify for particle transfer, as movement of these particles through TNTs is highly dynamic. This will help observing these particles when one or more stacks of a frame is blurred or unclear.

**Figure 5:**
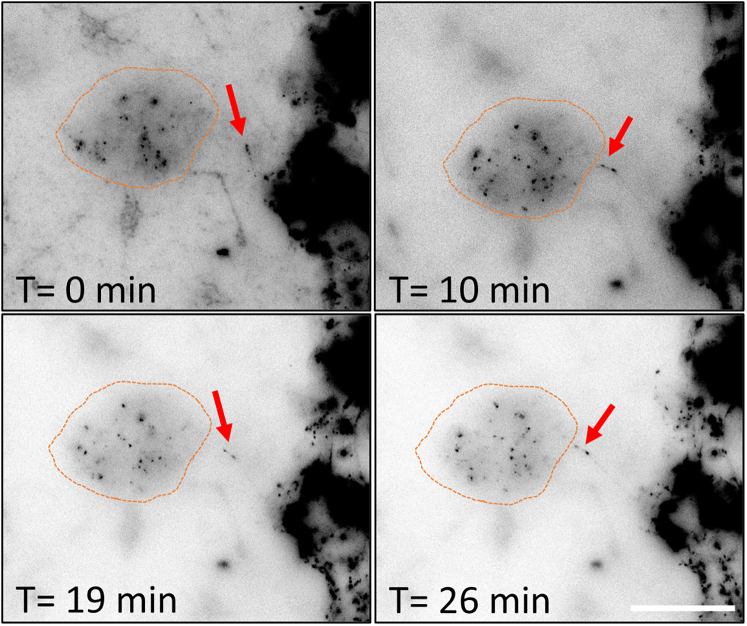
Example of transfer dynamic of α-synuclein through a TNT The red arrow points to α-synuclein inside a TNT, while the orange circle shows the microglia. α-synuclein is transferred from the neurons to the microglia during this time-lapse video. Scale bar is 10 μm.
  e. Use the tracking tool to allow for the tracking of fluorescent-labeled particles inside TNTs.
    i. From “Plugins” menu, select “Tracking”, then “Manual tracking”.
    ii. In the tracking window, change parameters to fit with the video properties being analyzed. For instance, time interval corresponds to the time (in min) for the duration of the video. The “x/y” calibration is the pixel size in μm, and “z calibration” corresponds to the number of stacks.
      ***Note:*** Information on video properties can be found from the “Image” menu, select “Properties”, or press Ctrl+Shift+P as a shortcut.
    iii. Select “Add track” then click on the particle of interest. It opens a new window that records the distance and velocity of specific particle.
    iv. The tracking tool moves automatically to the next frame of the video.
    v. Follow the particle by clicking on it until the end of the video.
    vi. Particle movement parameters are automatically recorded in the “Tracking” window.
      ***Note:*** It is possible to generate a line or dot drawing of the particle moving along the TNT, using the “drawing” option in the tracking window. It creates a new video that follows particle movement using a line.
9. Report the measurements of particles dynamics:
  a. Copy the values recorded using the tracking tool into an Excel file.
  b. Calculate the total travel distance (in μm) traveled by the particle, by summing all values in the “distance” column.
  c. Calculate the total velocity of particle transfer (in μm/min) by summing all values in the “velocity” column.
  d. Calculate the mean velocity (in μm/min) by averaging all values in the “velocity” column.
  e. Calculate the total time to travel from neuron to microglia by recording the time (min) the particle leaves the neuron until the time the particle arrives inside the microglia.

### Fixed cell analysis: Measuring TNT characteristics between neurons and microglia

**Timing: 1 h**

This procedure enables the measurement of TNTs characteristics between neurons and microglia. It also allows for the identification of α-synuclein or tau fibrils presence inside TNTs. The analysis can be performed using tools available in ImageJ software.

**Figure 6:**
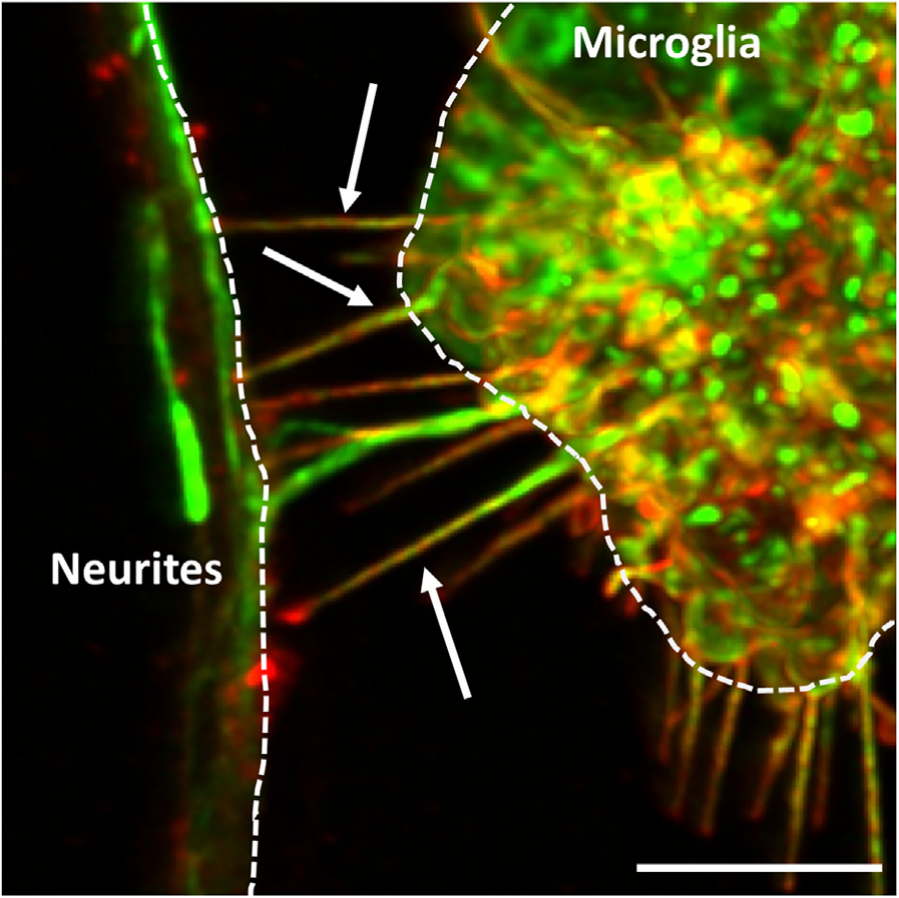
Immunostaining for TNTs between neurons and microglia Neurons and microglia were stained with Phalloidin (green) and WGA (red). White arrows point to TNTs between these cells, showing expression of both WGA and Phalloidin staining. Scale bar is 5 μm.
  e. Using the ROI manager tool (Analyze > Tools > ROI manager) and the “Freehand line” tool, draw a line starting from one hand of the TNT until the other end of the TNT.
  f. In the ROI manager, select “Add”, then “Measure”. This opens a “Results” window which records the length of TNTs and other values (Area, Mean, Min, Max, Angle).
  g. If more TNTs are measured in the same image, repeat the previous steps. The length of all TNTs will be recorded automatically to the results window and on the image (Figure 7).
    ***Note:*** The width of TNTs can also be measured and recorded using the same tools as described above. While a TNTs may be uniform along its length, a minimum of three points per TNT are measured to average its width – one in the middle and one for each end of the TNTs. It is also possible to count the number of TNTs, which can be performed manually using the cell counter option in Fiji.

10. TNT length and width measurement:
  a. Open image from immunostained microglia-neurons coculture in ImageJ.
  b. In the software options, select “Image” >“Stacks” > “Z Project”.
    ***Note:*** The ZProjection window opens, with the following sub-sections: “Start slice”, “Stop slice”, and “Projection type”. By default, these values are set to the first and last slices of the stacks taken during acquisition. In “Projection type”, we found the “Max Intensity” projection to be optimal for TNT identification.
  c. Click OK.
    ***Note:*** A maximum intensity Z-projection image will open in a new window, that merges all Z-stacks into one image, but allowing for visualizing every channels taken during acquisition separately.
  d. Adjust brightness and contrast as well as channels using the following options: Image > Adjust > Brightness/Contrast and Image > Color > Channels Tool.
    ***Note:*** TNTs must exhibit positivity for both F-actin and WGA to align with their definition. Therefore, verifying the colocalization of these markers is essential to confirm that the structure being measured is indeed a TNT. Furthermore, the TNT needs to sit above the substratum, which differentiate them with filopodia or neurites (Figure 6).

**Figure 7:**
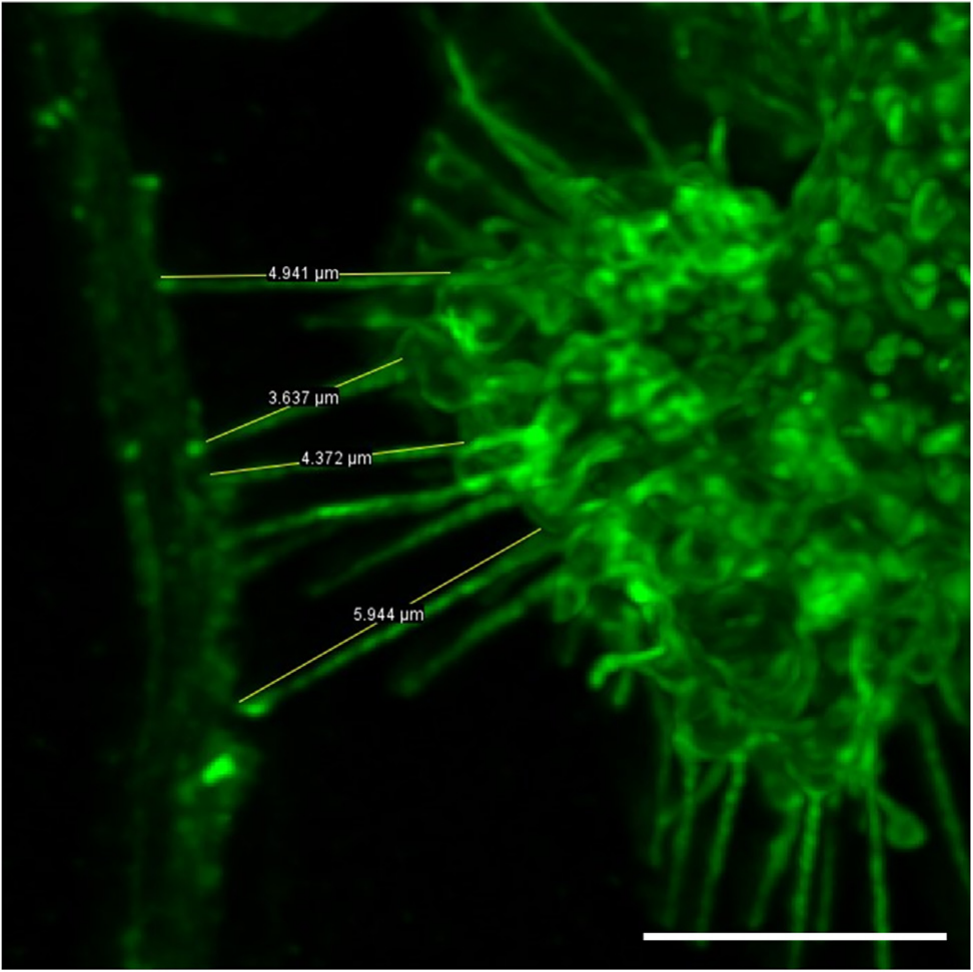
Example of TNT length measurement TNTs connecting neurons and microglia can be measured using the Freehand line option in ImageJ and using either the ROI management tool or the Ctrl+M option. Scale bar is 5 μm.
11. Measuring tube height above substratum (optional):
  a. In the ImageJ software, select “Image” > “Properties”.
  b. The image properties open in a new window. It provides the image properties, including voxel depth, which provides the spacing between z-slices. For example, a number of 1 voxel depth equal to 1 μm per z-slice.
  c. Using the voxel depth and scanning through each stack, it is possible to calculate the tube height above the substratum (Figure 8).

**Figure 8:**
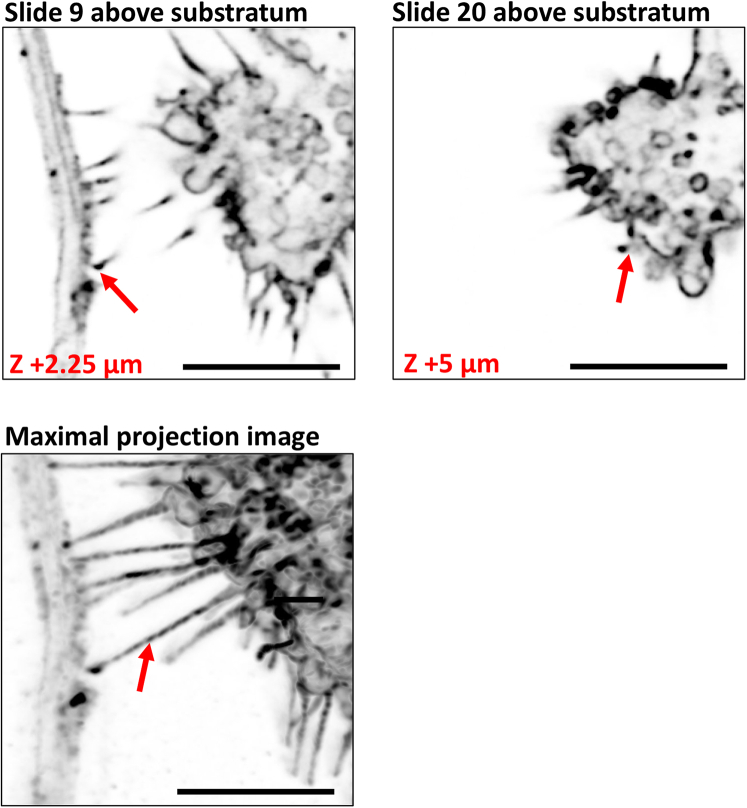
Example of TNT height above substratum The voxel size is 0.25 μm. In the stacks, the TNT start to appear at slide 9, which corresponds to + 2.25 μm above the substratum, and the TNT ends at slide 20, which corresponds to + 5 μm above the substratum. Maximal projection image shows the whole length of the TNT. Red arrows point to beginning and end of TNT, as well as whole TNT in maximal projection image. Scale bar is 5 μm.
12. Identification of -synuclein or tau fibrils inside TNTs (optional):
  a. In the ImageJ software, select “Image”>”Color”>”Channels Tool”.
  b. Set the α-synuclein or tau channel to gray scale.
  c. In “More”, select “Invert LUTs”.
    ***Note:*** This creates a black and white inverted image of your sample. It allows for better visualization of α-synuclein or tau particles.
  d. Scan through the different stacks of the image to identify for the presence of gray signal inside a TNT.
    ***Note:*** More than one particle may be visible inside the TNTs. The number of α-synuclein or tau particles within the TNT can also be quantified manually.

## Expected outcomes

By following the FACS protocol, it is possible to quantify the transfer kinetics of aggregated proteins (α-synuclein or tau) and organelles (mitochondria) between primary mouse neurons and microglia through tunneling nanotubes (Figure 3). This protocol can be adapted to quantify different organelles and proteins beyond aggregates and mitochondria as well as between different cell types and in-vitro systems.

Immunofluorescence protocol allows for visualization and quantification of TNTs that occur between cells with multi-dimensional characteristics such as lengths, diameters, heights, and the presence of aggregates as well as mitochondria inside TNTs (Figures 4, 5, 6, 7, and 8). Live imaging techniques help to capture the formation of TNTs in real time and provide additional information about TNTs’ transfer dynamics, including transfer velocity, transfer tracking, and TNTs’ persistence overtime (Figure 1). Similarly to the flow cytometry protocol, imaging protocols can be adapted in different ways and cell types to suit your scientific questions and goals.

## Limitations

The Flow Cytometry Protocol was designed and optimized to quantify aggregate transfers between neurons and microglia in a direct cell-to-cell manner, which is one of the main requirements for transfers via TNTs; reliable methods were also used to inhibit the formation of and transfers via TNTs either by mechanical inhibition or chemical inhibition of actin polymerization. However, due to a lack of TNT-specific markers and inhibitors, these methods have the potentials to affect other intercellular transfers that might rely on actin polymerization. Moreover, it cannot be determined with 100% certainty that the transfers recorded by flow cytometry are facilitated only by TNTs, as other cell-to-cell transfers might occur – i.e., phagocytosis, endocytosis, or gap junction transfer. It is therefore important to use adequate controls by testing drugs that inhibit these processes to make sure that any transfer observed is solely due to TNT transfer mechanism.

In live imaging, microglia and neurons may not form TNTs within the selected field of view. Therefore, it is essential to image different fields and repeat the experiment to obtain significant number of observations. Additionally, the stack settings during live imaging can impact TNT observations. While active formation of TNTs and transport of proteins or mitochondria TNT might occur, they can remain undetected due to suboptimal stack settings.

TNTs are thin cellular connections that extend above the substratum and closely resemble filopodia in shape and size, making it occasionally challenging to differentiate between the two. Additionally, the intricate structure of the neuronal network, with its extensive branching and neurites, can make it challenging to differentiate TNTs from these neuronal projections. An inadequate neuronal plating with denser cultures may render the analysis of TNTs between neurons and microglia challenging. It is therefore essential to plate neurons at a specific density (as stated in the “before you begin” section), and use appropriate cell labeling to better identify TNTs between neurons and microglia.

Prolonged exposure to light flashes can cause microglia to burst and TNTs to break, further complicating the observation of live TNT formation and transfer dynamics. Also, fluorescent dyes, such as CellTrace, or fluorescently labeled proteins used in this protocol are highly light-sensitive and prone to photobleaching during recordings. Physical stress may also be a limitation, in particular during immunohistochemistry, as repeated washes and movement of the culture dish can cause the TNTs to break.

## Troubleshooting

### Problem 1

Microglia and neurons fail to detach, leading to low numbers of cells available for flow cytometry (Related to Preparation 1: α-syn, tau, and mitochondria transfer experiments using flow cytometry).

### Potential solution

• Longer incubation of cells with Accutase (up to 10 min) on a shaker on low rpm to speed up the detachment process.
• Gently scratch the wells with a P1000 pipet tip and gently flush the wells to detach the cells.

### Problem 2

The desired cell population cannot be found while running flow cytometry sample (related to Flow cytometry analyses).

### Potential solution

Due to difference in size between your neurons and your microglia, the voltages for FSC and SSC for the two-cell population might differ, and the desired cell population will not appear in your FSC-A and SSC-A display and cannot be selected if the wrong PMT voltages are used.

Extra numbers of controls, including unstained and single stained microglia and neurons should both be used in order to determine the correct PMT voltages for your cell types.

### Problem 3

Low signal from CellTrace Violet (Related to Flow Cytometry Analysis).

### Potential solution

CellTrace Violet staining only occurs in the absence of FBS whereas MitoTracker Red can be diluted and used with media containing FBS.

• Wash cells with PBS before staining with CellTrace Violet to remove traces of FBS.
• In case of co-staining with CellTrace Violet and MitoTracker Red, use PBS only to dilute stock solutions.

### Problem 4

No TNTs are forming in the field of view (related to preparation two: microglia-neuron coculture for live imaging).

### Potential solution

Select a new field of view or create a tile scan for multiple fields of view. Increasing the number of fields will maximize the chance of catching the formation of TNTs.

### Problem 5

Poor TNT labeling (related to preparation three: immunostaining for TNT observation).

### Potential solution

• Increase the time of incubation with CellTrace or WGA, and increase the wash off time to reduce the signal-to-noise ratio.
• Keep the labeling solution and labeled cells in the dark and minimize the time these cells are exposed to light.
• Adjust light source of the microscope to avoid photobleaching.
• Refer to the manufacturer’s manual for dyes reconstitution, storage and protocol for adequate labeling.

### Problem 6

TNTs are broken (related to fixed cell imaging analyses).

### Potential solution

Take extra care when handling the stained culture, especially when moving location. Even small vibrations can cause the TNTs to break. The fixation solution indeed render the TNTs more fragile than during live imaging.

### Problem 7

Problem at observing protein movement (related to live imaging analyses).

### Potential solution

• Increase the brightness and/or contrast to observe dimmer or smaller fluorescent particles.
• Inverting LUTs may also help in visualizing these particles.
• Scan through every stack and through the whole video for particle movement.

## Resource availability

### Lead contact

Further information and requests for resources and reagents should be directed to and will be fulfilled by the lead contact, Michael T. Heneka (michael.heneka@uni.lu).

### Technical contact

Questions about the technical specifics of performing the protocol should be directed to the technical contacts, Vivian Baker (vivian.baker-hoernicke@uni.lu) and Dimitri Budinger (dimitri.budinger@uni.lu).

### Materials availability

This study did not generate new unique reagents.

### Data and code availability

This paper does not report original code.

Any additional information required to reanalyze the data reported in this paper is available from the lead contact upon request.

## Acknowledgments

The graphical abstract was created using Biorender.com. This work was supported by funding from the Deutsche Forschungsgemeinschaft (DFG, German Research Foundation) under Germany’s Excellence Strategy (EXC2151-390873048) to M.T.H. and the Fonds National de la Recherche within the PEARL program (FNR/16745220) to M.T.H. The authors also thank Dr. Paul Antony at the LCSB Bioimaging for microscope training and advice and Olga Kondratyeva at the LCSB Bioimaging Platform for cell sorting assistance. We would like to thank Dr. Ronald Melki for generously donating the fluorescent fibrils. We would also like to thank Dr. Hannah Scheiblich, who described TNT formation function between neurons and microglia and is the first author of the original manuscript on which this protocol is based.

## Author contributions

V.B., D.B., and M.T.H. designed the study. V.B. and D.B. performed experiments and generated primary data. V.B. and D.B. performed data analysis. S.-P.R. and M.T.H. supervised data analysis and experiments. All authors reviewed the final version of the manuscript.

## Declaration of interests

M.T.H. serves as an advisory board member at IFM Therapeutics, T3D, and Alector.
